# Light Mitigates Bismuth Toxicity While Sustaining Iron Homeostasis in *Lepidium sativum* Seedlings

**DOI:** 10.3390/plants15121898

**Published:** 2026-06-18

**Authors:** Cristina Caissutti, Davide Marzi, Giuseppe Capobianco, Silvia Serranti, Valerio Licursi, Massimo Zacchini, Patrizia Brunetti

**Affiliations:** 1 Research Institute on Terrestrial Ecosystems-National Research Council (IRET-CNR), 00010 Rome, Italy; cristinacaissutti@cnr.it (C.C.); massimo.zacchini@cnr.it (M.Z.); patrizia.brunetti@cnr.it (P.B.); 2 Department for Innovation in Biological, Agro-Food and Forest Systems, University of Tuscia, 01100 Viterbo, Italy; 3 NBFC, National Biodiversity Future Center S.c.a.r.l., 90133 Palermo, Italy; 4 Department of Chemical Engineering, Materials and Environment, Sapienza University of Rome, 00184 Rome, Italy; giuseppe.capobianco@uniroma1.it (G.C.); silvia.serranti@uniroma1.it (S.S.); 5 Institute of Molecular Biology and Pathology-National Research Council (IBPM-CNR), c/o Department of Biology and Biotechnology “C. Darwin”, Sapienza University of Rome, 00185 Rome, Italy; valerio.licursi@cnr.it

**Keywords:** abiotic stress, garden cress, gene expression, heavy metals, phytotoxicity

## Abstract

Bismuth (Bi) is increasingly used as a substitute for lead (Pb) in several industrial applications, raising concerns about its potential environmental impact. However, the effects of Bi on early plant development and nutrient homeostasis remain poorly understood. In this study, toxicological and transcriptional responses were investigated in garden cress (*Lepidium sativum* L.) exposed in vitro to increasing Bi concentrations (0, 30, 60, 121, and 242 mg L^−1^) under dark and light conditions. In darkness, Bi progressively reduced the germination index and root growth. In contrast, under light conditions, low Bi concentrations stimulated seedling growth, whereas this effect decreased at higher doses. Gene expression analyses showed that Bi differentially affected key genes involved in iron (Fe) uptake and homeostasis, including *LsIRT1* and *LsFRO2*, which displayed divergent expression patterns in dark- and light-grown seedlings. Micro-X-ray fluorescence (µ-XRF) analysis revealed distinct Fe and Bi accumulation profiles under dark and light conditions. Moreover, *LsPCS1* expression, a marker of heavy metal detoxification responses, was strongly induced in the shoots of light-grown seedlings, where Bi accumulation was detected. Taken together, these results show that Bi inhibits early seedling development in darkness by impairing Fe uptake and homeostasis, whereas light promotes tolerance to Bi by enhancing these processes.

## 1. Introduction

Bismuth (Bi) is widely regarded as a relatively low-toxicity heavy metal and is increasingly used in metallurgy, pharmaceuticals, cosmetics, and lead-free technologies [1]. This expanding use is expected to increase its release into terrestrial and aquatic environments, yet its biological impact on plants remains poorly resolved. A recent review emphasized that knowledge of Bi–plant interactions is still fragmentary, despite evidence that Bi can be taken up and translocated, impairing growth, genome integrity, and primary physiological processes [1]. Recent studies have demonstrated that Bi exposure elicits toxic effects in garden cress (*Lepidium sativum* L.), which takes up and accumulates the metal and exhibits morphological, physiological, and genotoxic alterations [2,3]. In plants, one of the few mechanistic frameworks currently available suggest that Bi toxicity is closely linked to the disruption of iron (Fe) homeostasis [4,5]. In higher plants such as *Arabidopsis thaliana* and garden cress, Fe acquisition in roots depends on the Strategy I system, which involves rhizosphere acidification, reduction of Fe^3+^ by Ferric Reduction Oxidase 2 (FRO2), a membrane-bound ferric chelate reductase, and uptake of Fe^2+^ by Iron-Regulated Transporter 1 (IRT1), a divalent cation transporter [6]. In addition, Ferric Reduction Oxidase 3 (FRO3) also participates in cell Fe homeostasis [7]. This pathway is tightly regulated because Fe is essential for cellular metabolism but potentially toxic when deregulated. In Arabidopsis, Bi impairs early seedling development, primarily at the root level, inhibiting primary root elongation and lateral root formation, while perturbing Fe homeostasis through the induction of *IRT1* expression, ultimately resulting in Fe overaccumulation and altered localization in roots [4,5]. Furthermore, Bi exposure may activate detoxification mechanisms commonly involved in the response to non-essential metals, including glutathione-dependent pathways and the synthesis of phytochelatins (PCs) mediated by phytochelatin synthases such as Phytochelatin Synthase 1 (PCS1), thereby promoting the chelation and sequestration of toxic ions [8]. Light also plays a key role in modulating plant responses to metal stress, influencing both metal translocation and detoxification mechanisms. In particular, Fe homeostasis is tightly interconnected with light signaling, as light regulates the expression of key components of the Fe-deficiency response and promotes shoot-to-root communication controlling Fe uptake and allocation [9]. Furthermore, several components of the photosynthetic machinery, activated by light, require Fe as a cofactor [9]. In parallel, PCs biosynthesis depends on glutathione availability and plant redox status, linking metal detoxification to redox metabolism, which is especially active in photosynthetic tissues [10]. However, only limited studies have investigated the molecular effects of Bi in plants. These gaps are particularly relevant in garden cress, a standard species for phytotoxicity testing and an excellent model for early seedling responses [2,3]. In this context, micro-X-ray fluorescence (µ-XRF) represents a valuable analytical tool for investigating metal-associated changes in elemental composition and distribution in plant tissues; therefore, it could provide information on Bi accumulation and impairment of Fe homeostasis [11].

In this study, Bi toxicity was investigated in garden cress through an integrated ecotoxicological, molecular, and physiological approach to uncover whether increasing Bi concentrations could affect germination, early seedling growth, Fe homeostasis, and metal detoxification responses under dark and light conditions. Since light strongly influences seedling development, Fe uptake, and stress tolerance, we hypothesized that different light conditions could modulate Bi toxicity and tolerance by altering physiological and molecular responses. Putative homologs of candidate genes involved in Fe homeostasis and metal detoxification were identified in garden cress, and their expression profile was evaluated under Bi exposure in dark- and light-grown seedlings. In parallel, Bi-induced changes in Fe uptake and Bi accumulation were assessed through µ-XRF. Altogether, this study unveils novel insights into the physiological and molecular bases of Bi toxicity in garden cress seedlings, highlighting the role of light in Bi stress responses and tolerance.

## 2. Results and Discussion

### 2.1. Root Growth Responses to Bismuth Are Modulated by Light/Dark Exposure in Garden Cress Seedlings

**Figure 1:**
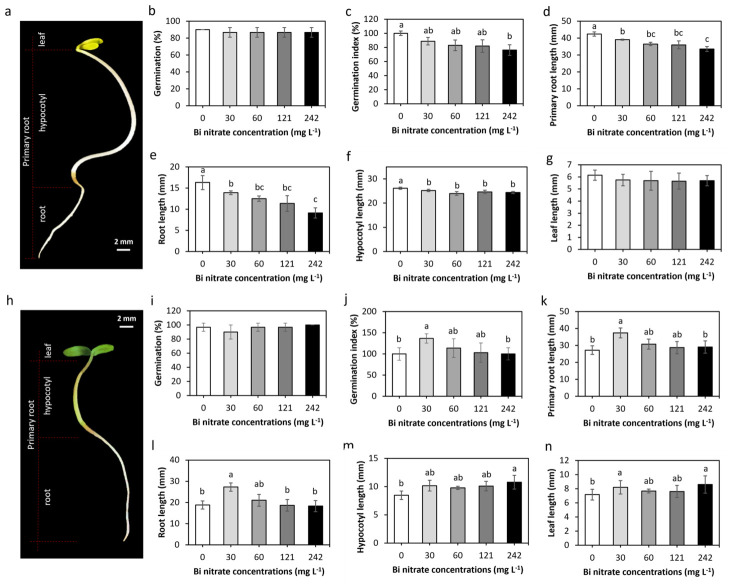
Analysis of bismuth (Bi) toxicity in garden cress (*Lepidium sativum* L.). Different morpho-physiological parameters were evaluated in dark-grown garden cress seedling (**a**) exposed to increasing Bi nitrate concentrations, including seed germination percentage (**b**), germination index (**c**), primary root length (**d**), root length (**e**), hypocotyl length (**f**), and leaf length (**g**). Likewise, in light-grown seedlings (**h**) the same parameters were evaluated (**i**–**n**). Data presented are mean ± standard deviation (n = 3). Statistically significant differences were evaluated through one-way ANOVA followed by Tukey’s test (*p* ≤ 0.05) and are indicated by different letters.

Plant responses to bismuth (Bi) were evaluated applying standard phytotoxicity assays using garden cress (*Lepidium sativum* L.) seedlings grown in vitro on moistened paper under increasing Bi concentrations [12]. In dark-grown seedlings, germination was not affected by any of the Bi concentrations tested. However, the germination index (%), which integrates seed germination percentage and primary root elongation, was slightly reduced at 30, 60, and 121 mg L^−1^ Bi relative to the control (0 Bi), and significantly reduced at 242 mg L^−1^ Bi (Figure 1a–c).

Consistent with this result, primary root length, which includes the root and hypocotyl, was significantly reduced at 30, 60, and 121 mg L^−1^ Bi, and decreased further at 242 mg L^−1^ Bi compared with the control (Figure 1d). To better define the inhibitory effect of Bi on seedling growth, root length was analyzed separately and showed a significant reduction at 30, 60, and 121 mg L^−1^ Bi, decreasing even more at 242 mg L^−1^ Bi (Figure 1e). Likewise, hypocotyl length was slightly but significantly reduced at all Bi concentrations tested (Figure 1f), whereas no significant effect was detected on leaf length (Figure 1g). Overall, these results demonstrate that, under dark conditions, Bi exerts a clear inhibitory effect on early seedling development, with root growth representing the primary and most sensitive target of Bi toxicity. The selective impairment of root growth, together with the absence of detectable effects on leaf development, indicates that Bi predominantly interferes with root elongation rather than with overall seed germination or shoot expansion at this stage. These findings are consistent with previous reports describing similar inhibitory effects of Bi on germination index and primary root growth in garden cress [2]. To comprehensively characterize plant responses to Bi, the assay was also conducted under light conditions (Figure 1h–n). Germination percentage remained unaffected across all treatments, whereas the germination index increased significantly at 30 mg L^−1^ Bi, then progressively declined with increasing Bi concentration, reaching values comparable to the control at 242 mg L^−1^ Bi (Figure 1i,j). Consistent with this trend, primary root length was significantly increased at 30 mg L^−1^ Bi and gradually declined as Bi concentration increased (Figure 1k). This stimulatory effect was mainly attributable to a significant increase in root length at 30 mg L^−1^ Bi, whereas hypocotyl length showed a significant increase only at 242 mg L^−1^ Bi (Figure 1l,m). Interestingly, leaf length also showed a slight but significant increase at 30 and 242 mg L^−1^ Bi, compared to the control (Figure 1n). Taken together, these results reveal that light modulates the biological response to Bi, shifting its effect from inhibitory in darkness, to stimulatory at low doses under illumination. This pattern is consistent with hormetic responses, indicating that at subtoxic levels Bi promotes seedling growth in a light-dependent manner, an effect absent in darkness and likely associated with distinct developmental, physiological, and molecular contexts.

### 2.2. Identification of LsCDC27B as a Reliable Housekeeping Gene in Garden Cress

The marked differences between dark- and light-grown seedlings prompted us to investigate whether the contrasting phenotypic responses to Bi were associated with distinct transcriptional regulation.

**Figure 2:**
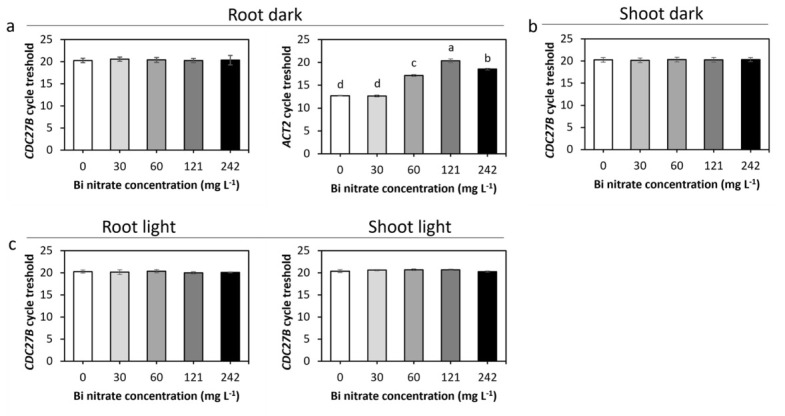
Reference gene evaluation in garden cress (*Lepidium sativum* L.). Evaluation of *LsCDC27B* and *LsACT2* expression stability in roots of dark-grown seedlings exposed to increasing Bi nitrate concentrations (**a**). Evaluation of *LsCDC27B* in shoots of dark-grown seedlings exposed to increasing Bi nitrate concentrations (**b**). Evaluation of *LsCDC27B* in roots and shoots of light-grown seedlings (**c**). Data presented are mean ± standard deviation (n = 5). Statistically significant differences were evaluated through one-way ANOVA followed by Tukey’s test (*p* ≤ 0.05) and are indicated by different letters.

Based on the literature, two candidate reference genes were identified in garden cress based on their homology to Arabidopsis: *ACT2*, related to structural cell functions, and *CDC27B*, involved in cell division and root apical meristem differentiation [13,14,15] (Table S2). Under increasing Bi concentrations in darkness, *LsACT2* expression in roots was highly variable and significantly upregulated at the highest doses, indicating that it is not a suitable housekeeping gene under these conditions (Figure 2a). By contrast, *LsCDC27B* expression remained stable in roots of dark-grown seedlings, as well as in shoots of dark-grown seedlings and in both roots and shoots of light-grown seedlings across all Bi concentrations, confirming its suitability as a reliable reference gene for subsequent analyses (Figure 2a–c). CDC27B plays a key role in columella development by sustaining the cell division activity required for root apical meristem function; indeed, loss of *AtCDC27B* impairs columella differentiation and results in a mitotically inactive meristem with markedly reduced amyloplast accumulation in Arabidopsis [15]. To further support the results on the stability of *LsCDC27B* expression under Bi treatment, Lugol’s staining was performed on roots from both dark- and light-grown seedlings to stain amyloplast in the columella cells (Figure S1a). The staining intensity and number of columella cells were similar across all Bi concentrations and controls in both dark- and light-grown seedlings, highlighting the absence of alterations that could be attributable to impairment in *LsCDC27B* expression, which was therefore identified as a stable, reliable housekeeping gene (Figure S1b–e).

### 2.3. LsFRO1, LsFRO2, and LsIRT1 Expression Is Oppositely Regulated in Dark- and Light-Grown Seedlings Exposed to Bismuth

As indicated in the literature, Arabidopsis seedlings treated with 2 μM Bi nitrate show increased expression of genes involved in iron (Fe) homeostasis, such as *AtIRT1*, and doubled Fe concentrations in roots compared to controls, while no differences are detected in the shoots, suggesting that Bi modulates the expression of genes involved in Fe absorption [4]. Homology analysis suggested that *AtIRT1* has two close homologs in garden cress, that we named *LsIRT1a* and *LsIRT1b*, which are coded by two different loci, whereas *LsFRO1* and *LsFRO2* were identified as putative homologs to *AtFRO3* and *AtFRO2*, respectively (Table S2). *LsFRO2* expression was significantly higher in light-grown seedlings, whereas *LsFRO1*, *LsIRT1a*, and *LsIRT1b* were significantly lower compared to dark-grown seedlings (Figure S2). According to the current model of Strategy I Fe uptake, Fe starvation, as well as light, promotes the expression of both *FRO2* and *IRT1*, whereas in darkness their transcription is lower; however, severe Fe deficiency can override light/dark regulation [9,16]. In addition, *IRT1* expression remains barely detectable in Arabidopsis seedlings up to three days after germination [17]. Thus, it is likely that during early development after germination, fine tuning of Fe homeostasis results in uncoupled *LsFRO2* and *LsIRT1* expression under dark and light conditions in garden cress.

**Figure 3:**
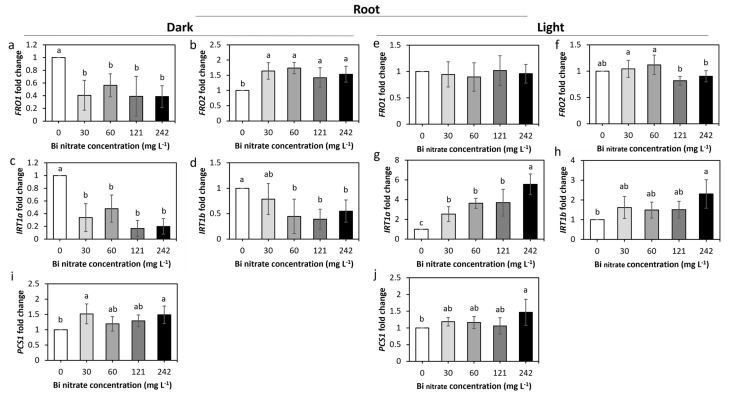
Expression of Fe homeostasis-related genes in garden cress roots (*Lepidium sativum* L.). *LsFRO1*, *LsFRO2*, *LsIRT1a*, *LsIRT1b* and *LsPCS1* in roots of dark- ((**a**–**d**), and (**i**), respectively) and light ((**e**–**h**), and (**j**), respectively)-grown seedlings exposed to increasing Bi nitrate concentrations. Data are presented as mean ± SD (n = 3). Statistical analysis was performed using one-way ANOVA followed by Tukey’s test (*p* ≤ 0.05); significant differences are indicated by different letters.

Several studies have demonstrated that exposure to high concentrations of non-Fe metals, such as nickel (Ni), cobalt (Co), and cadmium (Cd), induce *AtFRO2* and *AtIRT1* expression, resulting in either coordinated or uncoupled transcription of these genes [18]. So far, evidence for the impact of Bi on gene expression has been reported only for limited genes in Arabidopsis. Thus, to evaluate whether Bi also modulates the expression of putative homologs for *AtIRT1* as well as for *AtFRO3* and *AtFRO2* in garden cress, the transcription of *LsFRO1*, *LsFRO2*, *LsIRT1a* and *LsIRT1b*, was analyzed in both dark- and light-grown seedlings treated with increasing Bi concentrations. In roots of dark-grown seedlings, the expression of *LsFRO1* and *LsIRT1a* was significantly reduced at all Bi concentrations, whereas that of *LsIRT1b* was significantly reduced at 60, 121, and 242 mg L^−1^ Bi, compared to control (Figure 3a–d). Oppositely, *LsFRO2* expression significantly increased at 30 mg L^−1^ Bi compared to the control and remained at a similar level (~1.5-fold) across all Bi concentrations (Figure 3b). In contrast, in roots of light-grown seedlings, *LsFRO1* and *LsFRO2* expression was similar between Bi-treated and control plants (Figure 3e,f). *LsFRO2* showed slight but significant downregulation at 121 and 242 mg L^−1^ Bi compared to 30 and 60 mg L^−1^ Bi (Figure 3f). Conversely, *LsIRT1a* expression showed significant increase with increasing Bi concentrations, whereas *LsIRT1b* was significantly upregulated at 242 mg L^−1^ Bi (Figure 3g,h). These results indicate that Bi modulates the expression of genes involved in Fe uptake and homeostasis, in an almost opposite fashion under dark and light conditions during early seedling development. In detail, *LsFRO1* was repressed in dark-grown seedlings but remained unaffected in light, *LsFRO2* was induced in dark and unchanged in light, while *LsIRT1a* and *LsIRT1b* were repressed in darkness but induced in light. This regulation could be a strategy to limit Bi uptake in dark to prevent toxic responses, whereas Bi availability may induce *LsIRT1* expression in light to satisfy the request for cations required during photosynthetic processes. Among responses triggered by Bi, there is the activation of *PCS1*, which encodes phytochelatin synthase (PCs), a key enzyme involved in metal detoxification in plants [8]. Accordingly, *LsPCS1* expression was evaluated and found to be slightly but significantly increased in the roots of both dark- and light-grown seedlings relative to the respective controls, primarily at the highest Bi concentration, with a comparable magnitude under the two conditions (Figure 3i,j; Table S2). The induction of *LsPCS1*, to a similar extent, by Bi under both dark and light conditions supports the hypothesis that Bi elicits a conserved detoxification response by activating *PCS1* transcription . Similarly, the induction of *PCS1* expression by heavy metals in other plant species highlights its important role in detoxification responses [19].

**Figure 4:**
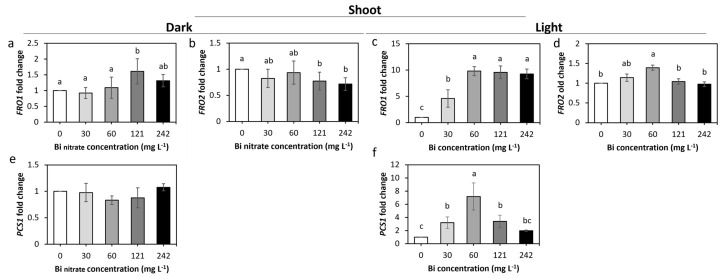
Expression of Fe homeostasis-related genes in garden cress shoots (*Lepidium sativum* L.). *LsFRO1*, *LsFRO2*, and *LsPCS1* in roots of dark- ((**a**,**b**,**e**), respectively) and light ((**c**,**d**,**f**), respectively)-grown seedlings exposed to increasing Bi nitrate concentrations. Data are presented as mean ± SD (n = 3). Statistical analysis was performed using one-way ANOVA followed by Tukey’s test (*p* ≤ 0.05); significant differences are indicated by different letters.

In Arabidopsis, Bi exerts negative effects mainly affecting roots, while in shoots they are negligible [4,5]. Thus, to determine whether a similar pattern occurs in garden cress, the expression of Fe-related genes was analyzed in both roots and shoots. According to the literature on *IRT1* in Arabidopsis, the expression of *LsIRT1a* and *LsIRT1b* was undetectable in shoots of dark- and light-grown garden cress seedlings [20]. *LsFRO1* was significantly upregulated at 121 mg L^−1^ Bi, whereas *LsFRO2* expression significantly decreased at 121 and 242 mg L^−1^ Bi, compared to control in dark conditions (Figure 4a,b). In shoots of light-grown seedlings, the expression of *LsFRO1* significantly increased at 30 mg L^−1^ Bi, and further showed a significant increase (~9 fold) at 60 mg L^−1^ Bi compared to the control, maintaining these high expression levels at 121 and 242 mg L^−1^ Bi (Figure 4c). *LsFRO2* showed a slight but significant increase at 60 mg L^−1^ Bi compared to control (Figure 4d). These results suggest that the induction of *LsFRO1* could occur to mitigate impaired Fe homeostasis, especially in light-grown seedlings. However, further studies are required to elucidate *LsFRO1* function in garden cress and Fe homeostasis. Interestingly, *LsPCS1* was unchanged in shoots of dark-grown seedlings, whereas a significant increase (up to ~7 fold) was observed in light-grown shoots at 30, 60, and 121 mg L^−1^ Bi, compared to control, suggesting the activation of detoxification responses under light conditions (Figure 4e,f). These findings further support the hypothesis that Bi induces a phytochelatin-mediated defense response, as reported for other toxic metals, such as Cd, arsenic (As), and lead (Pb), by modulating *LsPCS1* expression [8,9,10]. The peak observed at 60 mg L^−1^ Bi followed by a decrease at higher concentrations may indicate a biphasic response, likely reflecting a regulatory mechanism to prevent PCs overaccumulation, which is known to cause hypersensitivity or even toxicity in both Arabidopsis and rice (*Oryza sativa* L.) [21,22,23].

### 2.4. Bismuth Is Absorbed by Roots and Impairs Iron Homeostasis in Dark-Grown but Not in Light-Grown Seedlings

**Figure 5:**
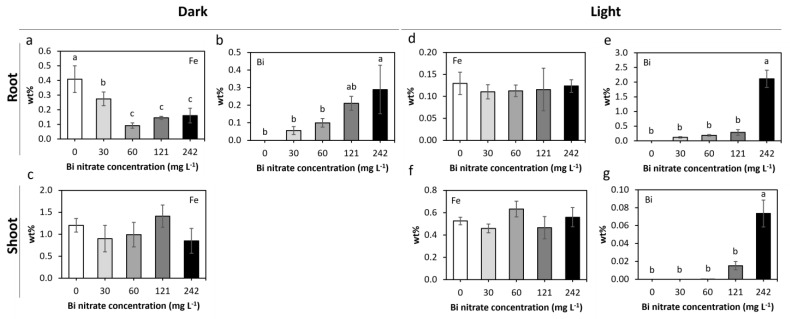
μ-XRF spectroscopic analyses. μ-XRF semi-quantitative elemental determination of Fe and Bi in roots ((**a**,**b**) respectively) and shoots (**c**) of dark-grown seedlings of garden cress (*Lepidium sativum* L.) exposed to increasing Bi concentrations. Bismuth was not detected in shoots of dark-grown seedlings. μ-XRF semi-quantitative elemental determination of Fe and Bi in roots ((**d**,**e**) respectively) and shoots ((**f**,**g**) respectively) of light-grown seedlings exposed to increasing Bi nitrate concentrations. Data presented are mean ± standard error (n = 3). Statistically significant differences were evaluated through one-way ANOVA followed by Tukey’s test (*p* ≤ 0.05) and are indicated by different letters. Mass percent, wt%.

Among recent technologies for metal detection in plant tissues, µ-XRF is emerging as a valuable alternative to conventional analytical methods because it enables rapid, non-destructive, multielement analysis with minimal sample preparation. Although quantification is based on X-ray fluorescence signals and fundamental-parameter calibration, µ-XRF has been shown to provide measurements that are highly correlated with ICP–OES data [11]. However, µ-XRF is a semi-quantitative technique that enables the detection of relative element accumulation; thus, changes in Bi and Fe accumulation across treatments were evaluated comparatively rather than in terms of absolute concentrations. According to *LsIRT1s* and *LsFRO1* expression, Fe concentration significantly decreased in roots of dark-grown seedlings treated with 30 mg L^−1^ Bi compared to the control, and decreased further at 60, 121, and 242 mg L^−1^ Bi (Figure 5a). Conversely, Bi accumulation increased progressively in roots exposed to increasing Bi concentration in the growth medium (Figure 5b). Iron concentration in shoots of dark-grown seedlings did not show significant differences across Bi treatments and control, while Bi was not detected (Figure 5c). In roots of light-grown seedlings, Fe levels remained similar across all Bi concentrations and the control, whereas Bi accumulation significantly increased at 242 mg L^−1^ Bi (Figure 5d,e). Similarly, in shoots of light-grown seedlings, Fe amount remained similar between Bi-treated and control seedlings, whereas Bi significantly accumulated at 242 mg L^−1^ Bi (Figure 5f,g). These results show that, although to limited extent, light promotes Bi translocation from root to shoot.

Altogether, our data suggests that Bi affects Fe homeostasis in roots of dark-grown seedlings by decreasing *LsFRO1*, *LsIRT1a* and *LsIRT1b* expression, whereas in light-grown seedlings the increase in *LsIRT1a* and *LsIRT1b* transcription may help in sustaining Fe homeostasis under Bi exposure. Concurrently, the accumulation of Bi in roots under both dark and light conditions suggests that Bi uptake could be mediated by transporters distinct from *LsIRT1*. Moreover, Bi induced the expression of *LsPCS1* in roots of both dark- and light-grown seedlings to similar extent, suggesting the activation of a conserved stress response. Iron amounts remained similar across all Bi treatments in shoots of both dark- and light-grown seedlings, respectively, whereas Bi was detected only in the shoots of light-grown seedlings. In this context, the upregulation of *LsFRO1*, and to lesser extent *LsFRO2*, could be a strategy to maintain Fe homeostasis under Bi accumulation. In addition, the *LsPCS1* was upregulated in shoots of light-grown seedlings, but not in dark-grown ones. These results indicate that, when accumulated, Bi induces *LsPCS1* expression. Bismuth translocation to shoots could be due to enhanced metabolic activity and redox processes induced by light in photosynthetic tissues, which require different cations as co-factors. The mobilization of cations during photosynthesis could further promote the expression of *LsPCS1* to limit potential damage in cells. Indeed, it is well established that chloroplasts are major sources of reactive oxygen species (ROS) under stress, and ROS act as key signaling molecules triggering detoxification pathways, including PC biosynthesis [24]. The lack of detectable Bi in shoots under darkness could be due to reduced translocation via the transpiration stream, as xylem transport is largely driven by transpiration, which is minimal in the absence of light [25]. Interestingly, results on Fe homeostasis slightly differ from those obtained in garden cress seedlings grown in soil, in which Bi reduced Fe accumulation in shoots, while Fe levels in roots remained unaffected [3]. These differences are likely to be caused by the different developmental stages of the plants analyzed. However, according to the literature, Bi was accumulated by both roots and shoots of garden cress seedlings [2,3].

Collectively, our work provides novel insights into the effects of Bi during plant development, expanding current knowledge on the regulation of Fe-related genes putative homologs in dark- and light-grown garden cress seedlings in response to this metal. These findings suggest that light-induced tolerance to Bi in garden cress likely relies on the modulation of Fe-responsive gene expression, which may regulate Fe homeostasis in roots and shoots. Additionally, our results show that Bi elicits the expression of *LsPCS1*, which probably activates PCs-mediated responses to limit Bi toxicity. Taken together, our data suggests that light promotes a fine regulation of Fe homeostasis during early seedling development, coupled with detoxification responses to overcome the negative effects exerted by Bi. Future studies using mutant lines for Fe-related genes, analyzing protein stability and enzymatic activities, will shed light on the precise mechanism regulating Bi tolerance. Further investigations into the interaction between light signaling and Fe uptake during the early phases of seedling development will unveil key information to improve metal stress tolerance during seed germination.

Overall, our findings identify light as a key factor shaping Bi responses in plants. In particular, the evidence that light enhances Bi translocation to shoots provides the basis for future studies aimed at clarifying whether the regulation of photosynthetic pathways can improve Bi phytoextraction efficiency. This may have relevant applications in phytoremediation, including the possible selection of plant species or genotypes with greater light-use efficiency, and testing whether these traits are associated with increased Bi accumulation. The specific activation of detoxification responses suggests that Bi tolerance depends on the coordinated interplay between metal transport, nutrient homeostasis, and chelation-based defense mechanisms. A deeper understanding of the processes underlying early seedling responses to metals, such as Bi, could support the development of strategies aimed at improving plant establishment under metal stress, as early growth represents a vulnerable developmental stage. Altogether, these results provide a basis for mechanistic investigations on Bi–plant interactions and for exploring their potential use in Bi monitoring and phytoremediation applications.

## 3. Materials and Methods

### 3.1. Germination Test and Seedling Growth Evaluation on Filter Paper

Seeds of garden cress (*Lepidium sativum* L.), purchased from Ingegnoli Spa (Milan, Italy), were used to evaluate the effects of bismuth nitrate [Bi(NO_3_)_3_·5H_2_O; Sigma-Aldrich, St. Louis, MO, USA] on seed germination and seedling growth. The assay was performed in 9 cm diameter plastic Petri dishes containing a single sheet of filter paper moistened with 5 mL of deionized water supplemented with 0 (control), 30, 60, 121, or 242 mg L^−1^ Bi nitrate, with three replicates per treatment, according to concentration indicated in the literature [2,3]. Ten seeds were placed in each Petri dish and incubated for 72 h at 25 ± 1 °C under either complete darkness or a 16 h light/8 h dark photoperiod with a light intensity of 150 µmol m^−2^ s^−1^. At the end of the experiment, toxicity was assessed by analyzing the number of germinated seeds and the length of roots, hypocotyls, and leaves. Two endpoints were considered: percent germination index (GI%) and primary root elongation [2,12]. The germination index (GI) was calculated as the product of the mean number of germinated seeds at the end of the test and the mean primary root elongation:

GI = mean number of germinated seeds × mean primary root elongation

The percentage germination index (GI%) was calculated as the ratio between the germination index of each treated sample (GI_sample) and the germination index of the corresponding negative control (GI_control), multiplied by 100:

GI% = (GI_sample/GI_control) × 100.

In addition, the effects of Bi nitrate on seedling growth were evaluated by measuring individual lengths of roots, hypocotyls, and leaves, through image acquisition and use of ImageJ software (version 1.46r). Finally, roots and shoots were sampled for each treatment for subsequent analysis.

### 3.2. Total RNA Extraction and qRT-PCR Analysis

Total RNA was extracted from roots and shoots of garden cress seedlings using the Plant Total RNA Mini Kit (Geneaid Biotech Ltd., New Taipei City, Taiwan). Reverse transcription was then performed with the PrimeScript™ RT Reagent Kit with gDNA Eraser (Takara Bio, Shiga, Japan). SYBR Green-based quantitative PCR assays were carried out as previously described [26]. All expression analyses were performed using three biological replicates. Relative transcript levels were normalized against the garden cress homolog of *CDC27B* (*LsCDC27B*), coding for a subunit of the anaphase-promoting complex (APC). Putative homologs of the genes of interest were identified in garden cress by sequence similarity searches against the available genomic scaffolds using BLAST (Basic Local Alignment Search Tool) through the Phytozome Plant Comparative Genomics portal (https://phytozome-next.jgi.doe.gov/blast-search (accessed on 11 March 2026), “Brassicales Map Alignment Project, DOE-JGI, http://bmap.jgi.doe.gov/” accessed on 11 March 2026). *Arabidopsis thaliana* coding sequences (CDSs) and predicted protein sequences were used as queries. Candidate garden cress sequences were assigned based on sequence identity and the functional annotation available for the *Lepidium sativum* genome [27]. The corresponding garden cress CDSs were retrieved using the JBrowse tool in Phytozome (https://phytozome-next.jgi.doe.gov/jbrowse, accessed on 11 March 2026) and used for primer design. The primers used in this study are listed in Table S1. The sequence homology between genes in *Arabidopsis* and garden cress are reported in Table S2.

### 3.3. Microscopic Observation of Starch Granules

Starch granules were visualized by staining with Lugol’s iodine (I_2_/KI) solution, following methods previously described [28]. Briefly, seedling roots were immersed in Lugol’s solution for 30 s, rinsed with deionized water, and mounted on microscope slides using a clearing solution containing chloral hydrate:glycerol:water (8:3:1, *v*/*v*/*v*). Images were acquired with an Axioskop 2 Plus microscope (Carl Zeiss, Oberkochen, Germany) equipped with an AxioCam ERc 5s camera (Carl Zeiss).

### 3.4. Micro X Ray Fluorescence (µ-XRF) Spectroscopy

X-ray fluorescence (XRF) spectroscopy analysis was performed using a μ-XRF benchtop spectrometer (M4 Tornado, Bruker^®^, Bruker Nano GmbH, Berlino, Germania) equipped with a Rh X-ray tube and XFlash^®^ detector (Bruker^®^), according to the literature [29]. Specific Areas in the Sample (SAS) were utilized to collect element spectra within plant samples (ESPRIT Bruker^®^ software, https://www.bruker.com/en/products-and-solutions/elemental-analyzers/eds-wds-ebsd-SEM-Micro-XRF/software-esprit-family.html, accessed on 11 March 2026). Spectrum energy calibration was performed daily using zirconium standard (ESPRIT Bruker^®^). The instrument chamber was set to 25 mbar, and samples were mounted between (0.01 mm) polyethylene films suspended in the chamber. A rhodium (Rh) X-ray tube operated at a constant excitation voltage of 50 kV and a current of 500 μA was used for all measurements. Semi-quantitative analysis was carried out using the Fundamental Parameter (FP) method, which relies on the theoretical relationship between X-ray fluorescence and sample composition previously described, and allows an accurate semi-quantitative analysis within complex plant matrices [30]. The factory-calibrated μ-XRF quantification method applies this principle with a correction based on a Bruker reference standard [31]. Data were pre-processed to highlight sample spectral differences and to reduce their variability, enabling accurate interpretation of the results obtained by the models. μ-XRF data were preprocessed to reduce matrix effects and improve signal quality using Standard Normal Variate (SNV), detrending, baseline correction, Savitzky–Golay smoothing, and mean centering, to enhance the linear relationship between signal intensity and element concentration [29,32].

### 3.5. Statistics

Statistical analyses were performed on data that met the assumption of normal distribution using one-way analysis of variance (ANOVA) in excel, as previously indicated [33]. When significant differences among treatments were detected, mean values were compared using Tukey’s multiple-comparison post hoc test. Differences were considered statistically significant at *p* ≤ 0.05. Data is presented as mean values of 3 or 5 biological replicates, with each biological replicate consisting of 10 seedlings. Different letters indicate statistically significant differences among treatments. When necessary, statistical analysis was performed using Student’s *t* test (* *p* < 0.05; ** *p* < 0.01; *** *p* < 0.001).

## 4. Conclusions

The growing demand for green technologies is driving the search for new materials such as bismuth (Bi), which is increasingly used in electronics, pharmaceuticals, cosmetics, and other low-toxicity industrial applications.

**Figure 6:**
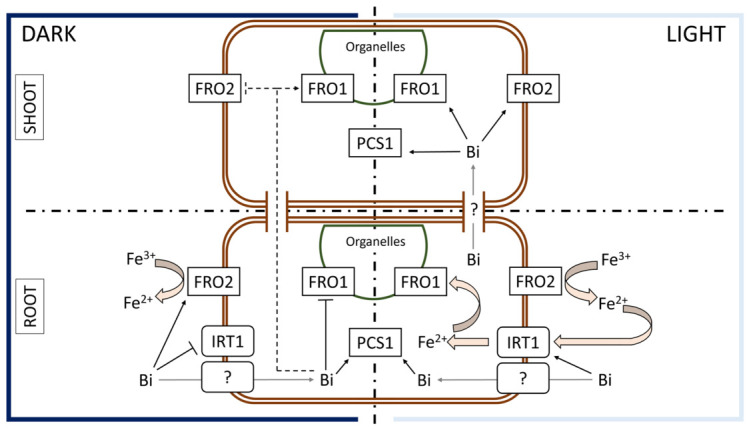
Speculative model of Bi effects in garden cress (*Lepidium sativum* L.). In roots of dark-grown seedlings, Bi represses *LsIRT1* and *LsFRO1* expression, while inducing *LsFRO2* and *LsPCS1*. Although not translocated to the shoots under these conditions, Bi affects shoot gene expression by repressing *LsFRO2* and inducing *LsFRO1*. In roots of light-grown seedlings, Bi induces *LsIRT1* and *LsPCS1*. Under light conditions, Bi is translocated to the shoots, where it induces *LsFRO1*, *LsFRO2*, and *LsPCS1* expression. Black arrows indicate gene induction, whereas black bar-headed arrows indicate gene repression. Black dashed arrows indicate changes in gene expression occurring in tissues where Bi translocation was not detected. Grey arrows indicate Bi transport. Question marks indicate unknown transporters.

However, the literature on Bi phytotoxicity is still scarce. To fill this gap, in this study we investigated toxicity and molecular responses in dark- and light-grown garden cress (*Lepidium sativum* L.) seedlings exposed to increasing Bi concentrations. In this context, a speculative model is proposed to summarize the main results obtained (Figure 6).

## Data Availability

The original contributions presented in this study are included in the article. Further inquiries can be directed to the corresponding author.

## Supplementary Materials

The following supporting information can be downloaded at https://www.mdpi.com/article/10.3390/plants15121898/s1, Table S1: List of primers used in this study; Table S2: Sequence identity of endogenous genes identified in *Lepidium sativum*; Figure S1: Analysis of columella in garden cress roots; Figure S2: Gene expression in dark vs. light grown garden cress.
