# Supplementary material for: Light Mitigates Bismuth Toxicity While Sustaining Iron Homeostasis in Lepidium sativum Seedlings
Source: Plants (Basel). 2026 Jun 18;15(12):1898. doi: 10.3390/plants15121898 (PMC13307138; doi:10.3390/plants15121898)
Supplement: Supplementary file 1 [file plants-15-01898-s001.zip › plants-4302749-supplementary.pdf]

Supplementary Materials

Table S1. List of primers used in this study.

Primer name	Primer sequence
LsCDC27B **For**	GGGACAGCTTTGCATGCCTTAAAGAGAA
LsCDC27B **Rev**	CCCATTAAAGCGTAAACGCTGCTCTCTG
LsACT2 **For**	AGCTGCTGGAATCCACGAGACCA
LsACT2 **Rev**	TCCTCTCGGGTGGTGCAACGA
LsIRT1a **For**	GTATCGGCTCCTCTCTTTAGCCAATACGTT
LsIRT1a **Rev**	GACAACATAGCGACAAATCCAGTGAAAGGG
LsIRT1b **For**	TTTAGTCGGTCAGTTCCATTCCTCCGAC
LsIRT1b **Rev**	TAGAGCAGCCAACATAGCCAGAAATCCA
LsFRO1 **For**	CTGCAGCAGCTACTATGGCAGTCCA
LsFRO1 **Rev**	AAGAACTCCCACGCTTGAACCCTCT
LsFRO2 **For**	TGGTCCTGCTTCTACCGATTTCTTGAGG
LsFRO2 **Rev**	TAATGGCAAGACAAGATCAAGCATGGCG
LsPCS1 **For**	ACCTCCTCAAACATGGTCAGGGAT
LsPCS1 **Rev**	ACAGCTGAAGTTGGCGTCGAA

Table S2. Sequence identity of endogenous genes identified in *Lepidium sativum*. Percent sequence identity at the gene, coding DNA sequence (CDS), and protein (amino acid) levels between the corresponding homologs in *Lepidium sativum* and *Arabidopsis thaliana*.

**Genes in *Arabidopsis thaliana***	**Homologous sequences in *Lepidium sativum***	**Gene identity**	**CDS identity**	**Protein identity**
CDC27B *(AT2G20000)*	*Lesat.1022s0076 (**LsCDC27B**)*	65.16%	92.98%	95%
ACT2 *(AT3G18780)*	*Lesat.0019s0329 (**LsACT2**)*	63.03%	94.44%	100%
IRT1 *(AT4G19690)*	*Lesat.0027s0091 (**LsIRT1a**)*<br>*Lesat.0101s0302 (**LsIRT1b**)*	40.01%<br>51.79%	63.67%<br>83.87%	75%<br>88%
FRO2 *(AT1G01580)*	*Lesat.0042s0065 (**LsFRO2**)*	58.55%	80.80%	85%
FRO3 *(AT1G23020)*	*Lesat.0042s0064 (**LsFRO1**)*	53.64%	76.14%	76%
PCS1 *(AT5G44070)*	Lesat.0027s0190 (***LsPCS1***)	54.19%	90.67%	92%

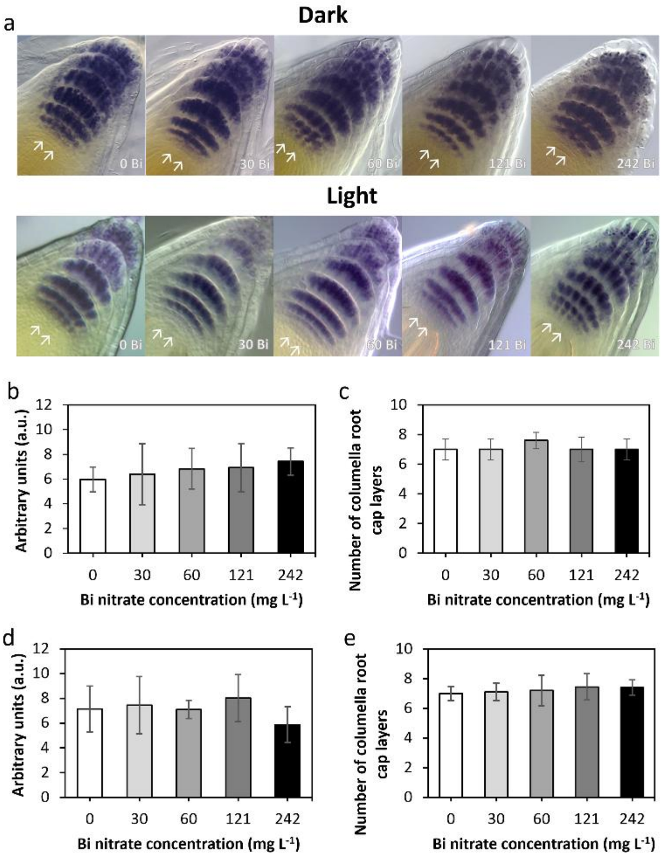

Figure S1. Analysis of columella in garden cress roots. Lugol's staining was used to visualize starch-rich columella cells of the root cap in roots of dark and light grown seedlings (upper and lower panel, respectively) exposed to increasing Bi nitrate concentrations (a). Lugol's staining intensity evaluation and count of columella root cap layers in dark grown (b, c) and light grown (d, e) seedlings exposed to increasing Bi nitrate concentrations. Data presented are mean ± standard deviation (n=3). Statistically significant differences were evaluated through one-way ANOVA followed by Tukey's test ($p \leq 0.05$) and are indicated by different letters.

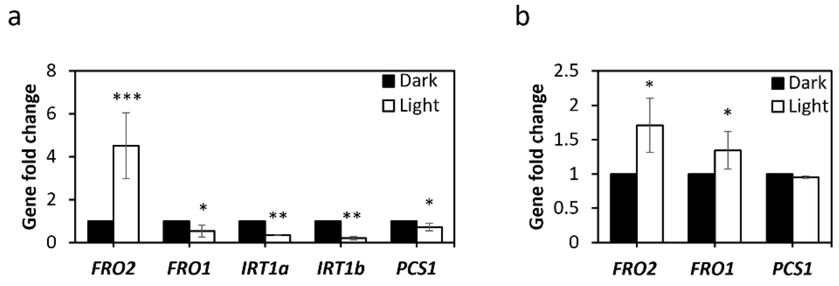

Figure S2. Gene expression in dark vs light grown garden cress. *LsFRO2*, *LsFRO1*, *LsIRT1a*, *LsIRT1b* and *LsPCS1* expression in roots of dark-grown seedlings compared to light-grown seedlings (a), and in shoots (b). Gene expression fold changes were calculated by normalizing expression levels to those detected in dark-grown seedlings for each gene. Asterisks indicate statistically significant differences calculated using the Student's T test (* $p < 0.05$; ** $p < 0.01$; *** $p < 0.001$).
